# A machine learning predictive model based on conventional two-dimensional echocardiography and serum biomarkers for early detection of ascending aorta dilation in BAV patients

**DOI:** 10.3389/fcvm.2026.1734730

**Published:** 2026-02-26

**Authors:** Xingyu Long, Yunxia Niu, Guixuan Nie, Sijing He, Liping Cui, Lisha Na

**Affiliations:** 1 Department of Cardiac Function Examination of Heart Centre, General Hospital of Ningxia Medical University, Yinchuan, Ningxia, China; 2 Department of Hematology, General Hospital of Ningxia Medical University, Yinchuan, Ningxia, China; 3 Department of General Practice, General Hospital of Ningxia Medical University, Yinchuan, Ningxia, China

**Keywords:** ascending aorta dilation, bicuspid aortic valve, LASSO, machine learning, nomogram, predictive models

## Abstract

**Objective:**

In order to address the challenge of early detection of ascending aortic dilation (AAD) in patients with bicuspid aortic valve (BAV), a machine learning prediction model integrating ultrasound hemodynamics and serum markers was developed to break through the limitations of traditional anatomical indicators.

**Methods:**

A total of 51 patients with BAV were prospectively enrolled and divided into ascending aortic dilation group (BAV-D, *n* = 25) and non-dilated group (BAV-ND, *n* = 26). Two-dimensional echocardiographic parameters [ascending aorta maximum flow rate (AAoV), mean pressure difference (AAoMPG)] and blood lipid markers [High-Density Lipoprotein Cholesterol (HDL-C), ApoB, etc.] were collected, and the key predictors were screened by the Least Absolute Shrinkage and Selection Operator (LASSO) algorithm, and the logistic regression model was constructed and the nomogram was visualized. Leave one cross-validation (LOOCV) was used to evaluate the robustness of the model.

**Results:**

AAoV, AAoMPG and HDL-C in the BAV-D group were significantly higher than those in the BAV-ND group (all *P* < 0.05). LASSO screened out five core predictors: age, HDL-C, ApoB, left ventricular mass index (LVMI), and AAoV. The AUC of the model was 0.825 (95% CI: 0.694–0.933), the accuracy was 74.5% (sensitivity 72.0%, specificity 76.9%), and the nomogram verification AUC was 0.809.

**Conclusion:**

The machine learning model constructed by integrating hemodynamics (AAoV) and metabolic markers (HDL-C and ApoB) for the first time can accurately quantify the risk of AAD in BAV patients, and its performance is significantly better than that of a single anatomical parameter, providing a visual decision-making tool for early intervention.

## 1 Introduction

Bicuspid Aortic Valve (BAV) is the most common congenital heart disease, with a prevalence of 1%–2% in the general population (1, 2). The most serious complications are ascending aortic dilatation (AAD) and dissection, which is associated with proximal aortic dilation (called bicuspid aortopathy) in about 50% of patients with BAV, resulting in a significantly higher aorta-related mortality rate and an eight-fold increase in the risk of aortic dissection than in the general population (3, 4). Traditionally, the diameter of the aorta has been the mainstay of surgical intervention (recommended threshold 5.0–5.5 cm) (5). However, approximately 40% of BAV-associated dissections occur in patients with an aorta diameter <5.5 cm (4, 6), and anatomical markers alone are not effective in distinguishing the risk of disease progression between different phenotypes (e.g., root or tubular) (7, 8). This highlights the inadequacy of the current risk stratification strategy.

In addition, the pathological mechanism of BAV-related aortic lesions involves multifactorial interactions. First, in terms of genetic factors, mutations in genes such as NOTCH1 and GATA5 were significantly associated with root expansion, especially in young patients (9). Family studies have shown a 6.9-fold increased risk of aortic disease in first-degree relatives (10). Second, there is an abnormal hemodynamic, and the abnormal leaflet opening of BAV leads to a disordered distribution of wall shear stress (WSS) in the ascending aorta wall, especially in right-left fusion BAV, and 4D Flow Magnetic Resonance Imaging (MRI) confirms that the high WSS region is spatially consistent with elastic fiber degradation and differential expression of microRNA (such as miR-128-3p) (11–13). And the molecular pathway dysregulation of BAV-related diseases. Downregulation of endothelial nitric oxide synthase (eNOS) promotes the phenotypic switch of vascular smooth muscle cells toward a synthetic phenotype (14). The imbalance between matrix metalloproteinase (MMP-2/9) and tissue inhibitor (Tissue Inhibitor of Metalloproteinases-1,TIMP-1) leads to the degradation of the extracellular matrix, and MMP-2 is regionally heterogeneous in the aortic wall of BAV patients (15, 16). Oxidative stress is exacerbated: the plasma antioxidant markers (α-tocopherol, HDL-C) levels and inflammatory factors (C-Reactive Protein,CRP) are increased in BAV patients (17).

Although cyclic markers (e.g., MMP-2, TIMP-1, endotrophin) and imaging parameters (e.g., flow displacement index on 4D Flow MRI, WSS) have been proposed for risk stratification, there are the following limitations (12, 18). First, the predictive value of a single biomarker is limited (e.g., TIMP-1 is only negatively correlated with the Z-score of some aortic segments) (16); Second, the predictive performance of the traditional model for Tricuspid Aortic Valve (TAV)-related aortic aneurysm (AUC>0.8) was significantly better than that of BAV (AUC = 0.5–0.55). Third, there is a lack of individualized prediction tools that integrate clinical, imaging, and molecular multi-dimensional parameters (19).

In summary, this study is the first to combine clinical features (age), ultrasound hemodynamic parameters [ascending aortic maximum velocity (AAoV) and ascending aortic mean pressure gradient (AAoMPG)], and serum lipid markers (HDL-C and ApoB). By combining machine learning algorithms (such as LASSO) and Nomogram to construct a prediction model for ascending aortic dilation in BAV patients, it aims to break through the limitation of a single parameter and provide accurate decision-making tools for early surgical intervention.

## 2 Material and methods

### 2.1 Patient selection

This prospective cohort study recruited 51 consecutively admitted BAV patients at Ningxia Medical University General Hospital (January-November 2024). Ethical clearance was granted by the institutional review board (2020-280) with written informed consent obtained from all subjects. The diagnostic criteria for BAV adhere to contemporary international consensus guidelines. Patients were categorized into the BAV with ascending aorta dilation group (BAV-D, *n* = 25) and the BAV without dilation group (BAV-ND, *n* = 26) using standardized echocardiographic criteria for ascending aortic diameter. Exclusion criteria comprised: Smoking, alcohol consumption, the presence of cardiovascular clinical manifestations, hemodynamically significant valvulopathies (stenosis/regurgitation ≥ moderate), structural valve degeneration with calcification, previous cardiac surgical interventions, chronic cardiovascular conditions (including hypertension), metabolic disorders (dyslipidemia/diabetes mellitus), chronic kidney disease (stage ≥3), Takayasu Arteritis, and systemic comorbidities with end-organ failure. To minimize confounding, only patients with two BAV morphologies—right–left coronary cusp fusion and right–noncoronary cusp fusion—were included in this study. In the BAV-ND group, there were 18 patients with right–left fusion and 8 with right–noncoronary fusion; in the BAV-D group, there were 20 patients with right–left fusion and 5 with right–noncoronary fusion. Among the BAV-ND group, 13 patients (50%) were male, and in the BAV-D group, 13 patients (52%) were male.

Standardized baseline assessments comprising anthropometric measurements, blood pressure, and after 8-hour overnight fasting phlebotomy samples (5 mL) were systematically acquired from the study cohort. To ensure ethical considerations and maintain research integrity, this study was officially approved by the Research Ethics Committee of Ningxia Medical University General Hospital (Grant No. 2020-280).

### 2.2 Echocardiography

Standardized positioning protocols (left lateral/supine orientation) were implemented with synchronized respiratory control to minimize motion artifacts. A GE Vivid E95 echocardiography system (S5-1 probe) was used with the pre-set “Adult Echo” protocol for routine echocardiographic examination. Standard echocardiographic measurements included left ventricular dimensions (LVIDd) and wall thickness indices [Interventricular Septum thickness at end-diastole (IVSd), Left Ventricular Posterior Wall thickness at end-diastole (LVPWd)] assessed at end-diastole. Left atrial volumetric (LAV) quantification was performed via Simpson's biplane technique, with subsequent body surface area (BSA) normalization yielding the left atrial volume index (LAVI). Maximal ascending aortic diameter was quantified at a standardized measurement plane 3 cm distal to the sinotubular junction. Pulse Doppler was applied to acquire flow spectra of the ascending aorta at rest, and manual tracing was performed to obtain AAoV and AAoMPG of the ascending aorta.

### 2.3 Sample treatment

Blood processing involved 10-minute centrifugation at 3,000 rpm (4 °C) to isolate plasma components. Biochemical analyses were performed using a Hitachi 7180 automated biochemistry analyzer. Serum triglyceride (TG) quantification was performed enzymatically via the GPO-POD methodology; total cholesterol (TC) levels were determined via a CHOD-PAP assay kit. Atherogenic low-density lipoprotein cholesterol (LDL-C) and atheroprotective high-density lipoprotein cholesterol (HDL-C) were quantified via peroxidase-coupled enzymatic assays; apolipoprotein profiles [apolipoprotein A (ApoA)/apolipoprotein B (ApoB)] were determined through immunoturbidimetric quantification.

### 2.4 Machine learning model construction

One classification model based on patients' aortic valve morphology were established. Feature selection was performed using the Lasso algorithm via the glmnet package in R. Leave-one-out cross-validation (LOOCV) was additionally conducted to assess model robustness. Diagnostic discrimination was evaluated through ROC curve analysis implemented with the pROC library in R, and model performance was evaluated based on accuracy, sensitivity, and specificity. A nomogram was finally developed using the rms package to predict event occurrence.

### 2.5 Statistical analysis

Statistical analyses utilized SPSS 26.0 (IBM) with parametric continuous variables (mean ± SD) compared via independent t-tests, while nonparametric alternatives addressed skewed distributions. Diagnostic efficacy was quantified through ROC analysis (significance threshold: two-tailed *P* < 0.05).

## 3 Results

### 3.1 Comparison of baseline characteristics

Both groups of patients (BAV-D vs. BAV-ND) were comparable in terms of demographic characteristics, anthropometry, and hemodynamic parameters. As shown in Table 1, no statistically significant differences were shown in the inter-group comparisons of age, height, weight, BSA, Body Mass Index (BMI), Systolic Blood Pressure (SBP), and Diastolic Blood Pressure (DBP) (all *P* > 0.05).

**Table 1:** Comparison of characteristics between the Two groups (mean ± SD).

Features	BAV-D (*n* = 25)	BAV-ND (*n* = 26)	*F/t*	*P*
Age (years)	43.28 ± 9.81	38.53 ± 10.62	0.213	0.105
Height (cm)	168 ± 7.00	169 ± 7.91	0.242	0.836
Weight (kg)	66.50 ± 11.04	69.67 ± 15.28	1.037	0.401
BSA (kg/m^2^)	1.75 ± 0.16	1.79 ± 0.21	0.870	0.499
BMI (kg/m^2^)	23.27 ± 0.88	24.10 ± 3.52	3.757	0.555
SBP (mmHg)	121.32 ± 10.19	123.38 ± 14.19	3.757	0.555
DBP (mmHg)	83.40 ± 8.30	80.80 ± 8.92	1.460	0.288

BAV-D, bicuspid aortic valve with dilation; BVD-ND, bicuspid aortic valve without dilation; BSA, body surface area; BMI, body mass index; SBP, systolic blood pressure; DBP, diastolic blood pressure, SD, standard deviation.

### 3.2 Comparison of 2D-TTE measurements between groups

There were no statistically significant differences between the two groups (BAV-D and BAV-ND) in indicators of cardiac structure and function, including Left Atrial Area in Apical 2-Chamber View(LA-A2C),Left Atrial Area in Apical 4-Chamber View(LA-A4C), LA, LAVI, LVMI, and Ejection Fraction (EF) (all *P* > 0.05). See Table 2 for details. However, AAoV and AAoMPG were significantly higher in the BAV-D group compared with BAV-ND (AAoV:1.57 ± 0.44 vs. 1.29 ± 0.21 cm/s, *P* = 0.006; AAoMPG: 10.83 ± 6.80 vs. 6.76 ± 2.58 mmHg, *P* = 0.005).

**Table 2:** Comparison of 2D-TTE measurements between the two groups (mean ± SD).

Features	BAV-D (*n* = 25)	BAV-ND (*n* = 26)	*F/t*	*P*
LA-A4C (cm^2^)	13.91 ± 3.27	12.90 ± 2.77	0.806	0.239
LA-A2C (cm^2^)	14.25 ± 4.22	13.47 ± 3.29	2.201	0.465
LA (mL)	43.38 ± 15.41	38.46 ± 12.53	2.398	0.216
LAVI (mL/m^2^)	24.57 ± 7.73	21.42 ± 6.07	1.777	0.116
LVMI (g/m^2^)	84.08 ± 11.84	78.94 ± 10.07	1.553	0.101
EF (%)	66.56 ± 2.84	66.19 ± 2.60	0.128	0.632
AAoV (cm/s)	1.57 ± 0.44	1.29 ± 0.21	6.179	0.006*
AAoMPG (mmHg)	10.83 ± 6.80	6.76 ± 2.58	9.361	0.005*

BAV-D, bicuspid aortic valve with dilation; BVD-ND, bicuspid aortic valve without dilation; LA-A4C, Left Atrial Area in Apical 4-Chamber View; LA-A2C, Left Atrial Area in Apical 2, Chamber View; LA, left atrial volume; LAVI, left atrial volume index; LVMI, left ventricular mass index; EF, ejection fraction; AAoV, ascending aortic maximum velocity; AAoMPG, ascending aortic mean pressure gradient; SD: standard deviation.

* *P* < 0.05: the difference between groups is statistically significant.

### 3.3 Comparison of lipid biomarker levels between groups

The two groups of patients (BAV-D vs. BAV-ND) were comparable in lipid measures reflecting atherosclerosis risk. There were no statistically significant differences in the levels of TC, TG, LDL-C, ApoA, and ApoB between groups (all *P* > 0.05). Refer to Table 3 for further information. However, HDL-C levels were significantly higher in BAV-D compared to BAV-ND (1.25 ± 0.29 vs. 1.07 ± 0.26 mmol/L, *P* = 0.029).

**Table 3:** Comparison of Serum biomarker levels between groups (mean ± SD).

Features	BAV-D (*n* = 25)	BAV-ND(*n* = 26)	*F/t*	*P*
TC (mmol/L)	2.86 ± 1.64	3.26 ± 1.46	3.038	0.370
TG (mmol/L)	2.73 ± 1.67	2.66 ± 1.53	0.657	0.877
HDL-C (mmol/L)	1.25 ± 0.29	1.07 ± 0.26	0.104	0.029*
LDL-C (mmol/L)	2.63 ± 0.58	2.91 ± 0.93	1.580	0.208
ApoA (g/L)	1.36 ± 0.22	1.26 ± 0.21	0.340	0.095
ApoB (g/L)	0.76 ± 0.13	0.82 ± 0.19	1.381	0.154

BAV-D, bicuspid aortic valve with dilation; BVD-ND, bicuspid aortic valve without dilation; TC, total cholesterol; TG, triglyceride; HDL-C, high-density lipoprotein cholesterol; LDL-C, low density lipoprotein cholesterol; ApoA, apolipoprotein A; ApoB, apolipoprotein B; SD, standard deviation.

* *P* < 0.05: the difference between groups is statistically significant.

## 4 Feature selection and prediction model construction

### 4.1 Feature selection and model construction

**Figure 1:**
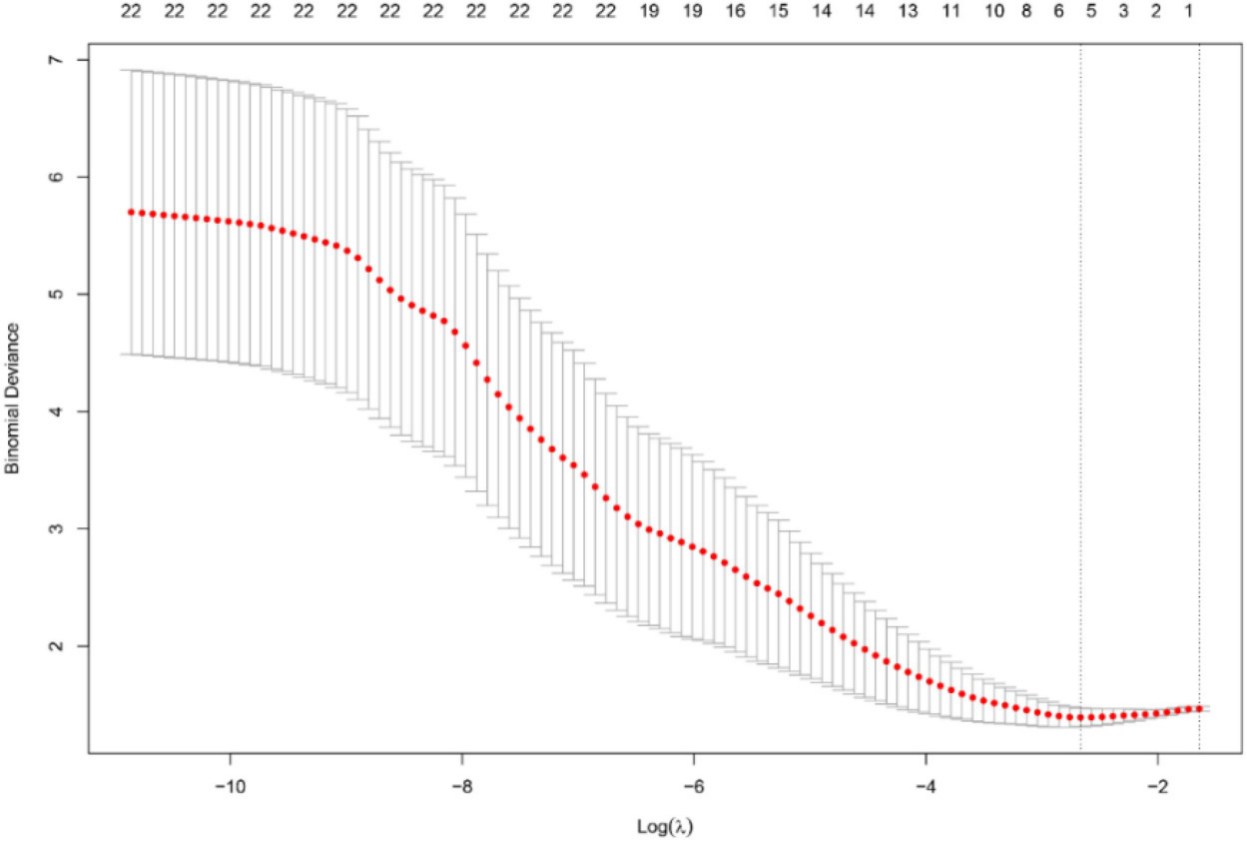
Feature selection process via lasso algorithm in R. The shrinkage path of each feature coefficient with the increase of log(*λ*) and the change of the mean binomial deviation (and standard deviation) of the 10-fold cross-validation with log(*λ*). The vertical dashed line indicates the optimal *λ* value selected based on the minimum deviation criterion, leaving five non-zero coefficient features.

The study extracted 22 potential predictive features from participants' clinical data, echocardiographic parameters, and serum biomarkers. In order to screen the most predictive features and avoid overfitting, the Lasso (L1 regularization) logistic regression model was used for feature selection. The regularization parameter *λ* is automatically optimized by 10-fold cross-validation (with the minimum Binomial Deviance as the criterion) and feature screening is completed (features with non-zero coefficients are retained). Figure 1 illustrates the trajectory of the Lasso coefficient path as a function of log(*λ*) and the cross-validation bias curve, with the optimal *λ* value (indicated by the vertical dashed line) filtering out the five predicted features (see Table 4). In order to evaluate the stability of the model, an additional Leave-One-Out Cross-Validation (LOOCV) was performed.

**Table 4:** Feature selection results.

Feature	Coefficients
Age (years)	0.1074
HDL-C (mmol/L)	0.2561
ApoB (g/L)	−0.1290
LVMI (g/m^2^)	0.1425
AAoV (cm/s)	0.4906

### 4.2 Model performance evaluation

**Figure 2:**
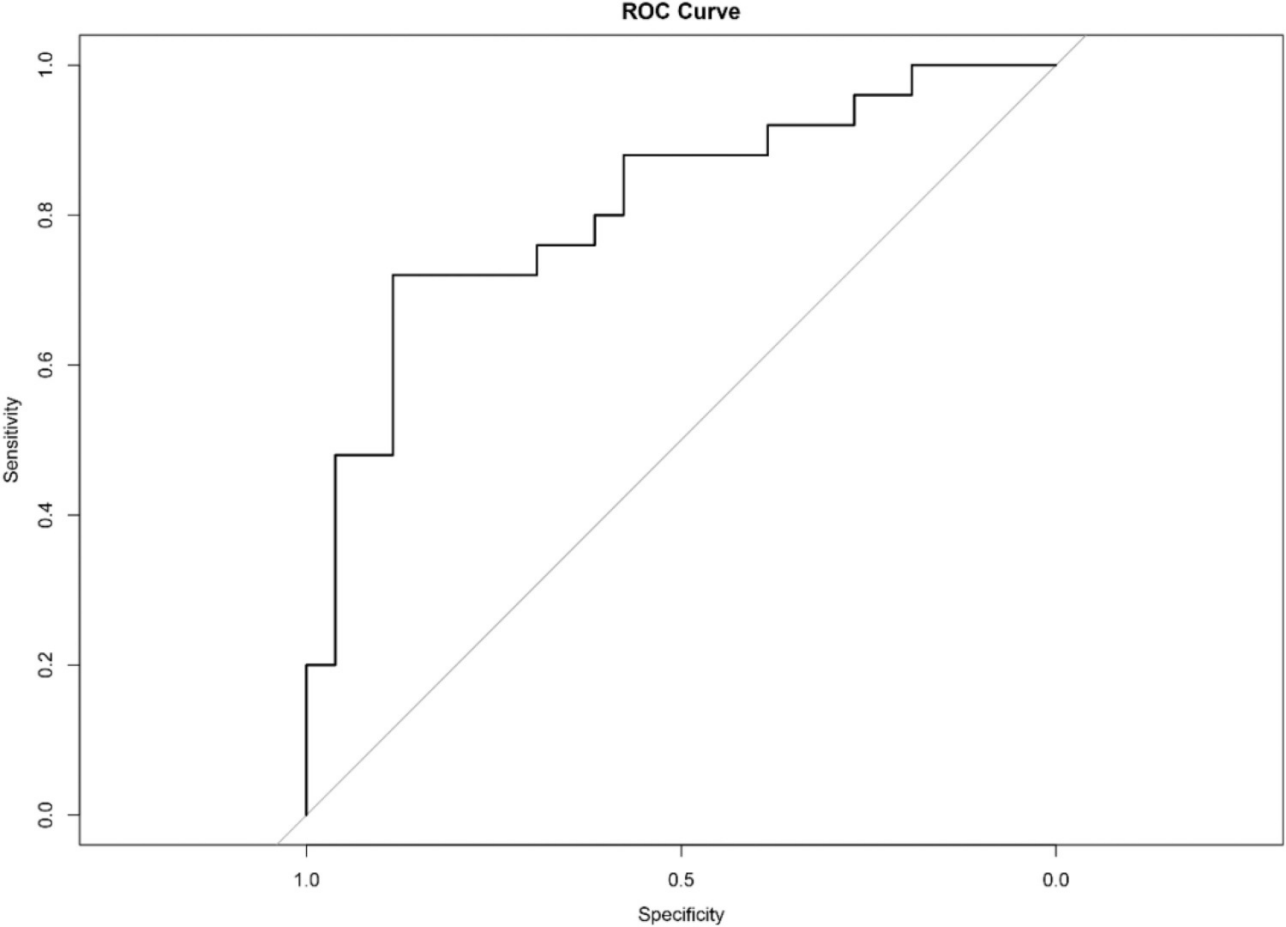
ROC curve of the lasso model. The curve illustrates the sensitivity of the model in relation to (1 - specificity) at different decision thresholds. The area under the curve (AUC) is 0.825, indicating that the model has good discriminant power. The diagonal line represents random guessing without the ability to discriminate.

Based on the selected features and their coefficients in Table 4, a Lasso logistic regression model was constructed to predict BAV-associated ascending aorta dilation. As shown in the ROC curve in Figure 2, the model demonstrated good discrimination in discriminating whether aortic dilation occurred or not, with area under the curve (AUC) = 0.825 (95% CI: 0.694–0.933). The model had an overall accuracy of 74.5% on the test set and exhibited a balanced sensitivity (72.0%) and specificity (76.9%). Additionally, we performed external validation of the model using bootstrapping as an additional assessment of model stability. The results showed a mean bootstrap-validated accuracy of 0.548 with a standard deviation of 0.111.

## 5 Construction and evaluation of individualized risk prediction nomogram

### 5.1 Nomogram construction

**Figure 3:**
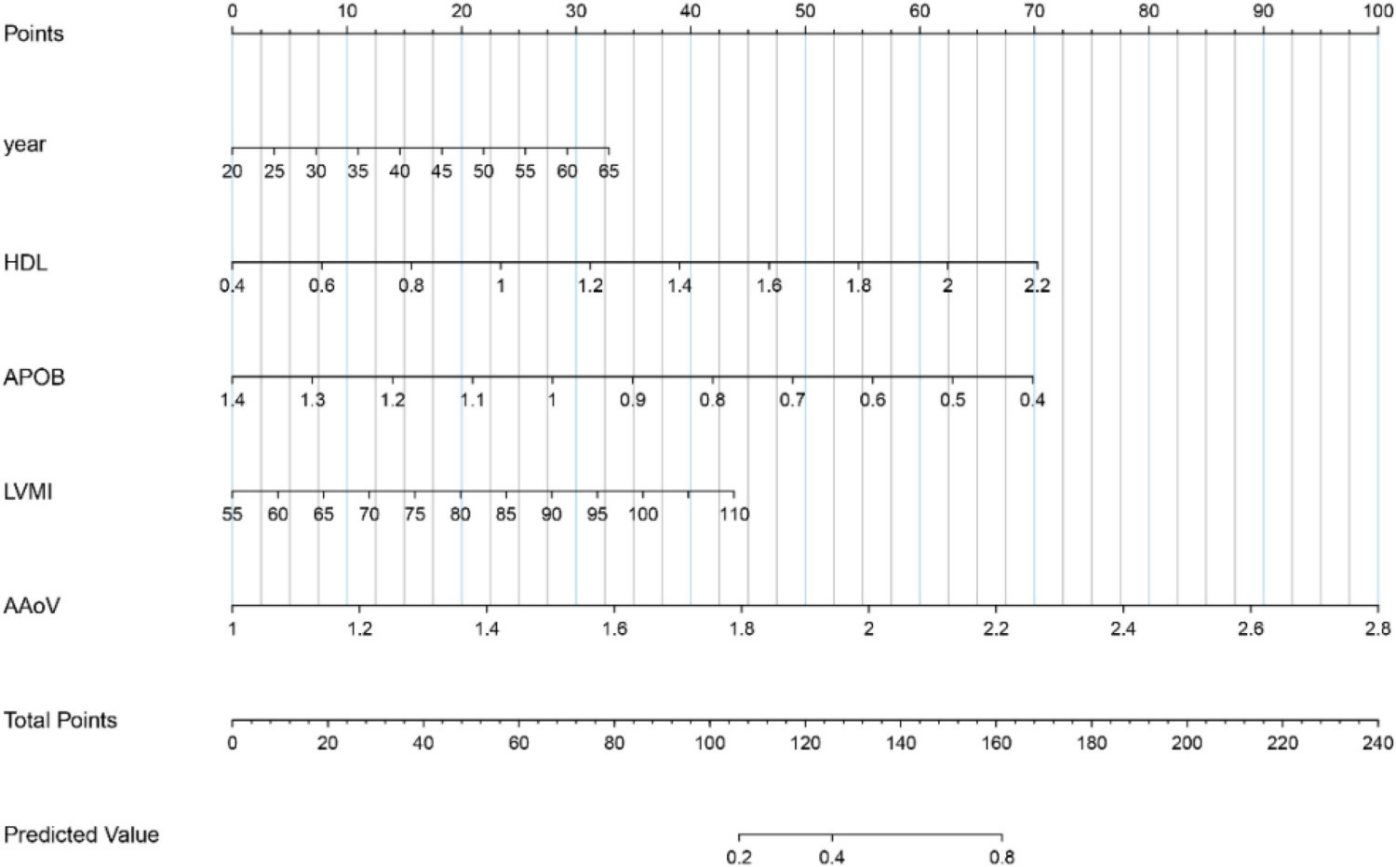
Nomogram for predicting BAV-associated ascending aorta dilation. This nomogram is used to visually predict the probability of ascending aorta dilation in individual BAV patients.

In order to facilitate clinicians to individualize and visualize the risk of ascending aorta dilation in patients with BAV, a nomogram was constructed using R software based on five independent predictors (age, HDL-C, ApoB, LVMI, AAoV) and their normalized coefficients (Table 4) screened by the Lasso model (Figure 3).

### 5.2 Nomogram performance verification

**Figure 4:**
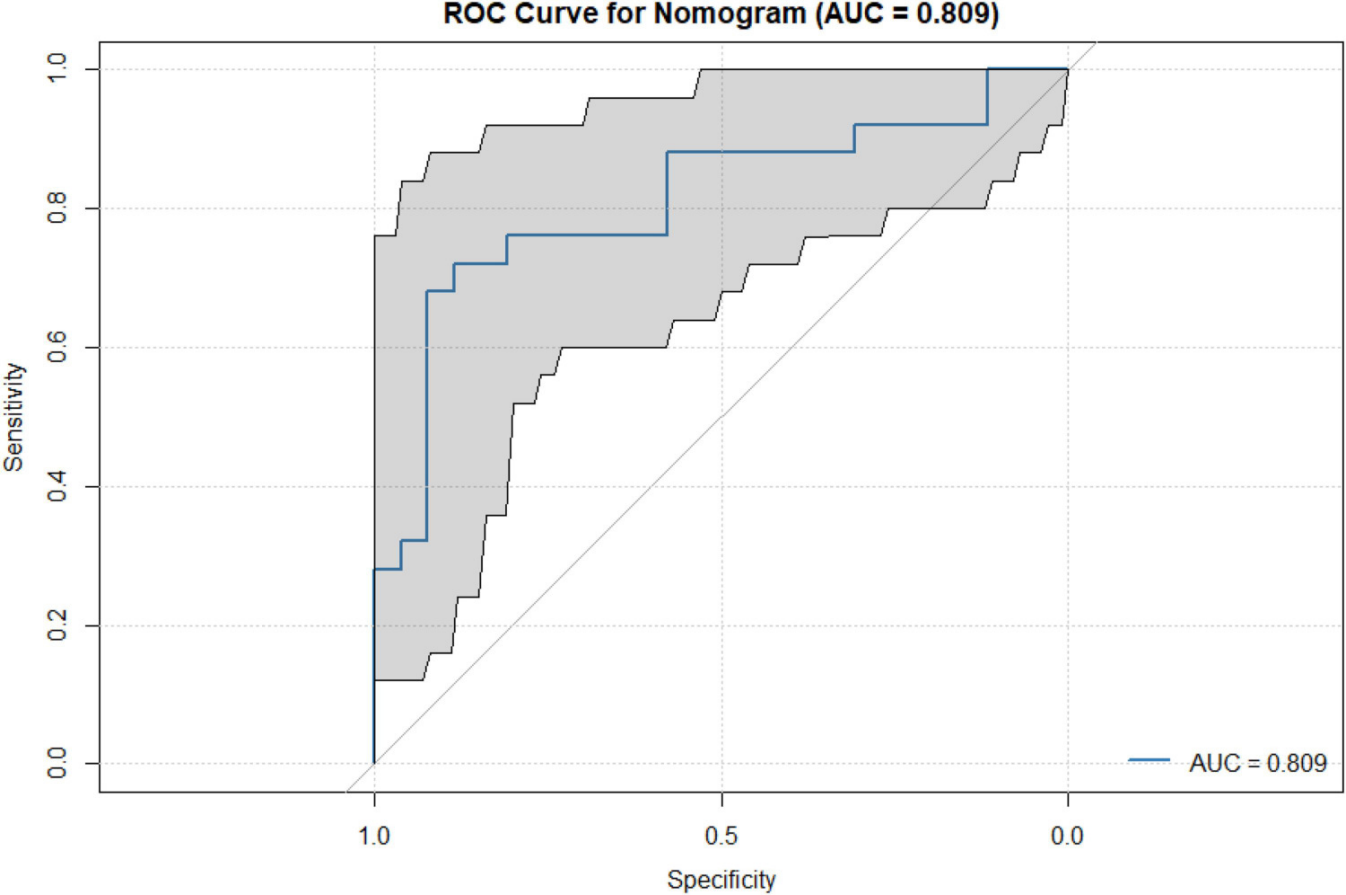
ROC curve for nomogram. This curve demonstrates the sensitivity and (1-specificity) relationship of the nomogram model in predicting BAV-related ascending aorta dilation at different decision thresholds. The area under the curve (AUC) is 0.809, indicating that the model has good discriminant power. The diagonal line represents random guessing without the ability to discriminate.

This nomogram model showed good discrimination in distinguishing whether ascending aorta dilation occurred in patients with BAV. As shown in the ROC curve of Figure 4, its area under the curve (AUC) = 0.809.

## 6 Discussion

In this study, a novel integrated method was established to combine hemodynamic indicators of 2D-TTE with serum lipid biomarkers for predicting ascending aortic dilation in patients with BAC. The nomogram based on LASSO algorithm showed good discrimination efficiency, highlighting the synergistic effect of abnormal hemodynamic stress and lipid metabolism disorder in BAV aortic lesions. Five key predictors: AAoV, HDL-C, ApoB, LVMI, and age were screened, which together translate complex pathophysiological interactions into clinically applicable risk assessment tools.

First, this study confirmed that AAoV and AAoMPG were significantly increased in the BAV plus ascending aortic dilation group, which was directly related to the unique blood flow pattern of BAV. Abnormal leaflet morphology of BAV (e.g., right-left coronary fusion) can produce eccentric jets that increase WSS in the ascending aorta wall, promoting endothelial injury and stromal remodeling (11, 20, 21). At the same time, the 4D Flow MRI study further showed that the abnormal flow displacement and WSS distribution of ascending aorta in BAV patients were positively correlated with the progression of aortic elasticity and dilation (22–24). This suggests that the AAoV and AAoMPG in our study have certain predictive value and also confirm the central role of hemodynamics in aortopathy.

Notably, HDL-C levels were significantly higher in the BAV-D group than in the non-dilated group, contrary to the traditional perception of cardiovascular risk. The possible mechanism of this phenomenon is that HDL-C may play a dual role by regulating oxidative stress and inflammatory response in aorta dilation in BAV patients. Studies have shown that patients with BAV have a systemic decrease in antioxidant capacity (e.g., decreased α-tocopherol) and inflammatory activation (elevated CRP), while HDL-C may be upregulated as a compensatory protective factor (1, 17). In addition, Martinez-Micaelo et al. found that the plasma antioxidant capacity of aorta dilation in BAV patients was significantly reduced, which was associated with HDL dysfunction (17). Thus, elevated HDL-C may reflect a compensatory response to oxidative damage in the blood vessels, rather than a mere protective marker.

The Lasso regression model constructed in this study integrated five parameters: age, HDL-C, ApoB, LVMI and AAoV, and its AUC reached 0.825, which was significantly better than that of a single anatomical index (such as aortic diameter). The Lasso model has three advantages to working with nomogram. First, the joint model covered multi-dimensional influencing factors, including hemodynamics (AAoV), metabolism (HDL-C and ApoB), myocardial remodeling (LVMI) and age factors, which fit the multifactorial pathogenesis of aorta dilation in BAV patients (8, 25, 26); Secondly, the joint model quantifies and concretizes the individualized risk assessment, and the Nomogram visualization tool quantifies the individual risk to assist clinical decision-making. For example, a 50-year-old patient with a score of 22 points for age, HDL-C of 1.2 mmol/L (30 points), ApoB of 1 g/L (28 points), LVMI of 80 g/m^2^ (20 points), and AAoV of 1.8 m/s (44 points) has a total score of 154 points, corresponding to a predicted risk probability between 0.4 and 0.8, approaching 0.8. This indicates a substantially elevated risk, warranting modifications in both the frequency and modalities of follow-up. Thirdly, the combined model can predict the occurrence and development of the disease at an early stage, and AAoV and LVMI may be abnormal before the aortic structure changes, providing a window for subclinical intervention (27).

In addition, our findings are consistent with those of Lopez-Sainz et al. quantifying abnormal blood flow in BAV by 4D Flow MRI compared to previous studies (12), but this study is the first to incorporate simple ultrasound parameters (AAoV and AAoMPG) into a prediction model, which is more applicable to grassroots outreach. At the same time, traditional studies have suggested that LDL-C promotes aortic atherosclerosis, but this study suggests that HDL-C and ApoB may be involved in aortic remodeling through non-atherosclerotic pathways (such as modulation of matrix metalloproteinase activity) in aorta dilation in BAV patients (28, 29). And machine learning predicted aortic dilation in TAV/BAV patients and found that the BAV group had lower predictive power (AUC 0.5–0.55), attributed to its stronger genetic dominance (19). However, the AUC of 0.825 in this study may be due to the fact that the study focused on BAV subsets and included hemodynamic markers. Compared with our model, their parameter selection relied solely on lipid biomarkers and failed to effectively integrate ultrasound-derived parameters—particularly the hemodynamic index AAoV, which was not selected as a key feature, thereby reducing the predictive performance of their model. This study is of great significance in clinical translation. First, the visual nomogram serves as a practical risk stratification tool. For instance, a hypothetical patient with a nomogram-derived total score > 150 (indicating high risk) might be recommended for more intensive surveillance, such as annual cardiac magnetic resonance imaging (MRI) to closely monitor aortic dimensions and wall stress. In contrast, a patient with a score < 100 (low risk) could potentially adhere to the standard biennial follow-up with transthoracic echocardiography. This individualized approach may optimize resource allocation and patient safety; Second, this study provides a clinical reference for the timing of clinical intervention. For example, early pharmacological intervention (e.g., statins) may be considered for patients whose aortic diameter is <4.5 cm predicted to be high-risk by the model (30); Third, complementary surgical decision-making, by combining the US guidelines (diameter threshold) and this model, can be individualized for patients with moderate dilation (4.0–5.0 cm) but high risk of the model (31).

This study has several limitations that should be acknowledged. First, the relatively small sample size constitutes a major limitation of this study; future external validation of the model in larger, multicenter cohorts is warranted. Furthermore, the resampling analysis based on bootstrapping, which yielded a reduced mean accuracy, underscores the limited generalizability of our findings and further diminishes the statistical power to detect subtle associations. Similarly, due to the limited sample size, the association between valve morphology and hemodynamics was not analyzed. Second, the analysis did not incorporate established genetic markers (e.g., *GATA5* mutations) or novel circulating biomarkers such as endotrophin (9, 18), which may provide deeper mechanistic insights and enhance risk stratification. Third, the absence of longitudinal data precludes an assessment of the model's predictive efficacy for tracking the progression of aortic dilation over time. Addressing these limitations in subsequent studies will be crucial for confirming the robustness and clinical utility of our results.

## Data Availability

The data analyzed in this study is subject to the following licenses/restrictions: Data supporting the results of this study may be obtained from the corresponding authors upon reasonable request. Requests to access these datasets should be directed to Lisha Na: lishana2003@163.com.
